# Complementary Nucleobase Interactions Drive Co-Assembly of Drugs and Nanocarriers for Selective Cancer Chemotherapy

**DOI:** 10.3390/pharmaceutics13111929

**Published:** 2021-11-15

**Authors:** Fasih Bintang Ilhami, Enyew Alemayehu Bayle, Chih-Chia Cheng

**Affiliations:** 1 Graduate Institute of Applied Science and Technology, National Taiwan University of Science and Technology, Taipei 10607, Taiwan; fasihilhami17@gmail.com (F.B.I.); enyewalemayehu@gmail.com (E.A.B.); 2 Advanced Membrane Materials Research Center, National Taiwan University of Science and Technology, Taipei 10607, Taiwan

**Keywords:** adenine–uracil base pair, complementary hydrogen bonded drug carrier system, controlled drug delivery, supramolecular nanogels, selective cytotoxicity

## Abstract

A new concept in cooperative adenine–uracil (A–U) hydrogen bonding interactions between anticancer drugs and nanocarrier complexes was successfully demonstrated by invoking the co-assembly of water soluble, uracil end-capped polyethylene glycol polymer (BU-PEG) upon association with the hydrophobic drug adenine-modified rhodamine (A-R6G). This concept holds promise as a smart and versatile drug delivery system for the achievement of targeted, more efficient cancer chemotherapy. Due to A–U base pairing between BU-PEG and A-R6G, BU-PEG has high tendency to interact with A-R6G, which leads to the formation of self-assembled A-R6G/BU-PEG nanogels in aqueous solution. The resulting nanogels exhibit a number of unique physical properties, including extremely high A-R6G-loading capacity, well-controlled, pH-triggered A-R6G release behavior, and excellent structural stability in biological media. Importantly, a series of in vitro cellular experiments clearly demonstrated that A-R6G/BU-PEG nanogels improved the selective uptake of A-R6G by cancer cells via endocytosis and promoted the intracellular release of A-R6G to subsequently induce apoptotic cell death, while control rhodamine/BU-PEG nanogels did not exert selective toxicity in cancer or normal cell lines. Overall, these results indicate that cooperative A–U base pairing within nanogels is a critical factor that improves selective drug uptake and effectively promotes apoptotic programmed cell death in cancer cells.

## 1. Introduction

The specific sequences present in biopolymers such as DNA, RNA, and proteins are responsible for the survival of complex, adaptable living organisms [1]. Analogous synthetic polymers with well-controlled, designed sequences have been predicted to function as components in a wide range of applications [2,3,4]. Complementary, noncovalent multiple hydrogen bonding interactions—such as adenine-thymine (A–T), guanine-cytosine, and adenine–uracil (A–U) base pairing between nucleic acids—provide versatile tools to control and tune the structure and function of polymers containing complementary nucleobases [5,6,7,8]. In recent decades, numerous research groups have reported that “bio-constituted” hydrogen bonding interactions could facilitate the self-assembly of various structures with nanometer-scale features. Moreover, well-controlled, dynamic physical properties and varied stimuli-responsive properties in response to environmental changes could be achieved by varying the amounts and strength of the nucleobase pairs [9,10,11]. For instance, supramolecular amphiphilic block copolymers with complementary adenine and thymine hydrogen bonding interactions that spontaneously self-assemble into nano-sized micelles in aqueous solution were reported by Kuang et al., and a well-controlled drug release profile could be obtained by tuning the content of A–T in the polymer structure [12]. Wang et al. designed a unique supramolecular phospholipid with moderate hydrogen bonding interaction recognition between adenosine and uridine; this phospholipid could self-assemble into spherical liposomes with highly pH-responsive ability, and had high potential for controlled drug release [13]. Fan and co-workers blended an adenine-containing amphiphilic block copolymer and uracil-functionalized crosslinking agent to self-assemble new micelles that exhibited rapid, pH-controlled drug release to significantly reduce cell viability [14]. Based on the examples above, synthetic supramolecular polymers containing nucleobase pairs are a promising concept in various areas of research, and the exploitation of non-covalent interactions to confer polymeric structures with dynamic characteristics may expand their applications.

The development of polymeric nanocarriers in order to improve chemotherapeutic efficacy and intracellular delivery for cancer therapy has attracted overwhelming enthusiasm in modern pharmaceutical technology. In terms of biocompatible nanocarriers, polyethylene glycol (PEG) is regarded as one of the most important components of nanocarriers for drug delivery, owing to its high level of water solubility, considerable chain mobility, and low toxicity, among other suitable physicochemical properties [15,16,17]. Moreover, the introduction of PEG segments into nanocarrier structures decreases their tendency to form aggregates, and thus results in enhanced structural stability and avoids the clearance of the nanocarriers by the reticuloendothelial system (RES) [18,19,20]. Koo et al. noted that shell cross-linked polymer micelles based on a combination of PEG and polyamino acids exhibited high stability in water, improved biocompatibility towards normal and cancer cells, and enhanced the intracellular release triggered by glutathione (GSH) [21]. Yokoyama et al. developed a new PEG-*b*-poly(α,β-aspartic acid) block copolymer; the resulting drug-loaded micelles were extremely stable in aqueous solution, and their size and distribution could be easily tuned [22]. Nevertheless, previous reports mentioned that not all hydrophilic PEG segments provide advantageous behavior in an aqueous environment [23,24,25]. A range of hydrophilic PEG segments have varied stabilization effects, including invoking side-effects via immunological responses, inhibiting the intracellular uptake of the nanocarrier, and non-biodegradability. Thus PEG segments with a molecular weight below 20 kDa are preferable for use in drug carriers [23,24,25,26,27]. Inspired by the specific features of nucleobases, we confidently speculated that the incorporation of nucleobase molecules into the terminal end-groups of PEG may confer unique self-assembly behavior and physical properties in aqueous solution, leading to potential candidates for drug delivery applications.

**Scheme 1:**
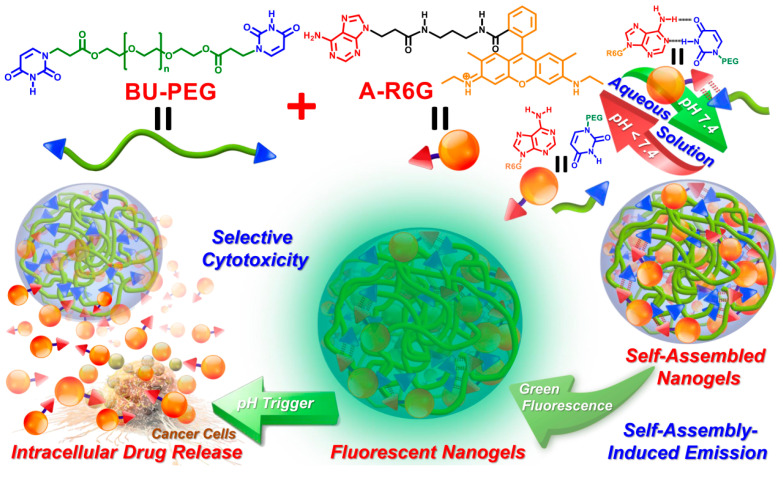
Schematic illustration of the co-assembly process, fluorescent properties, and cancer cell-selective cytotoxic behavior of hydrogen-bonded A-R6G/BU-PEG complexes. The upper-right green and red arrows represent the association and dissociation of the complementary A–U base pairs under various pH conditions.

A recent series of studies in our laboratory demonstrated that the introduction of complementary A–U base pairs within polymer structures confers the ability to spontaneously self-assemble into stable, physically crosslinked networks [28], resulting in excellent film-forming capability, tailorable mechanical performance and intriguing self-healing capacity upon tuning of the content of the A–U complexes in the matrix [29]. To further extend the concept of complementary nucleobase interactions to drug carrier systems, we herein design and synthesize a new difunctional, uracil-terminated PEG macromer (BU-PEG) that associates with adenine-functionalized rhodamine (A-R6G) in an aqueous environment via complementary A–U interactions, and thus results in the formation of self-assembled spherical nanogels with high structural stability (Scheme 1) [30]. In addition, the resulting A-R6G/BU-PEG nanogels possess a number of unique physical properties, including extremely high A-R6G-loading capacity, wide-range tailorable A-R6G-loading content, distinct green fluorescence behavior, and well-controlled pH-triggered A-R6G release. Importantly, a series of in vitro experiments clearly confirmed that the A-R6G-loaded BU-PEG nanogels highly selectively targeted cancer cells, and were selectively internalized by them, and could thus promote high levels of rapid apoptotic death through an endocytotic pathway, but they were not internalized by and did not harm normal cells. As far as we are aware, this is the first study to develop a complementary drug_carrier system based on hydrogen bonding interactions between stable A–U base pairs, with the goals of improving both the safety and effectiveness of cancer chemotherapy. Thus, this new concept for the fabrication of drug_carrier systems via complementary A–U base pairing offers in-depth insight into the benefits of manipulating the drug delivery behavior of nanocarriers with potential in various biological and biomedical applications.

## 2. Materials and Methods

### 2.1. Chemicals and Materials

Uracil (≥99% purity) and adenine (>99.5% purity) were purchased from Acros Organics (Geel, Belgium). Rhodamine 6G (R6G), polyethylene glycol (PEG, average molecular mass: 1900–2200 g/mol), dimethylformamide (DMF), potassium *tert*-butoxide (*t*-BuOK), triethylamine (TEA), deuterated chloroform (CDCl_3_), and deuterium oxide (D_2_O) were obtained from Sigma-Aldrich Chemical (Milwaukee, WI, USA) at the highest purity available. The HPLC-grade organic solvents were used as received from TEDIA (Fairfield, OH, USA). Phosphate-buffered saline (PBS), Dulbecco’s modified Eagle’s medium (DMEM), fetal bovine serum (FBS), penicillin-streptomycin, trypsin-EDTA, trypan blue, 4′,6-diamidino-2-phenylindole (DAPI), the Dead Cell Apoptosis Kit with Brilliant Violet-421™ Annexin V (BV421-Annexin V), and Ghost Dye™ Red 780 (GDR780) were purchased from Thermo Fisher Scientific (Waltham, MA, USA). All chemicals and reagents were employed as received. Adenine-functionalized rhodamine derivative (A-R6G) and PEG diacrylate (PEGDA, number average molecular weight (M_n_) = ~2000) were synthesized and characterized according to procedures that have been described previously [31,32,33].

### 2.2. Synthesis of BU-PEG

PEGDA (4 g, 2 mmol) and uracil (0.5 g, 4.5 mmol) were dissolved in 300 mL of DMF, and agitated for 48 h at 60 °C with a small quantity of *t*-BuOK as a catalyst (0.04 g, 0.003 mmol). After removing DMF by vacuum distillation, the crude product was dissolved in chloroform (200 mL) and the insoluble impurities were removed by filtration through a Büchner funnel. Subsequently, the chloroform was evaporated by rotary evaporation, then the obtained product was washed three times with diethyl ether and dried in an oven at 30 °C for 1 day. The yield was 83% (3.8 g).

### 2.3. Characterization

#### 2.3.1. Proton and Carbon Nuclear Magnetic Resonance (^1^H NMR and ^13^C NMR)

The ^1^H NMR and ^13^C NMR spectra were obtained on a Bruker AVIII NMR spectrometer (Billerica, MA, USA) at 500 MHz; the 25 mg samples were dissolved in 1 mL of CDCl_3_ and D_2_O, respectively.

#### 2.3.2. Gel Permeation Chromatography (GPC)

Molecular weight information was obtained using a Waters Alliance 2690 HPLC Separation Module (Waters Corporation, Milford, MA, USA) with tetrahydrofuran (THF) as the mobile phase at a flow rate of 1.0 mL/min and 40 °C. The *M*_n_ and the polydispersity index (PDI) were determined by comparison against a series of narrow distribution polystyrene standards.

#### 2.3.3. Critical Micelle Concentration (CMC)

Pyrene was utilized as a fluorescent probe to measure the CMC of the PEG and BU-PEG polymers. Varied concentrations of samples (0.00001 to 0.4 mg/mL) were prepared in water in advance. Next, 10 μL of pyrene solution was dropped into the tubes, then the mixtures were sonicated and incubated at 4 °C overnight to allow the polymers and aqueous phase to stabilize fully. All samples were examined using a fluorescence spectrometer (Jasco FP-8300 Spectrophotometer Hitachi, Tokyo, Japan) at an excitation wavelength of 335 nm. The emission intensities at 373 nm and 392 nm were recorded and plotted against the sample concentration in order to determine the CMC values.

#### 2.3.4. Particle Size and Surface Charge

Hydrodynamic particle size, PDI, and zeta (*ζ*) potential values were measured with a dynamic light scattering particle analyzer (DLS, Nano Brook 90Plus PALS, Brookhaven, Holtsville, NY, USA) connected to a 632-nm He-Ne laser beam (scattering angle: 90°). DLS measurements for each sample were repeated ten times, and averaged.

#### 2.3.5. Photoluminescence (PL) and Ultraviolet-Visible (UV-Vis)

The PL and UV-Vis spectra of samples in aqueous solution were acquired at 25 °C using Hitachi F4500 luminescence and Jasco FP-8300 spectrophotometers (Hitachi, Tokyo, Japan), respectively.

#### 2.3.6. Atomic Force Microscopy (AFM) and Scanning Electron Microscopy (SEM)

The surface morphologies of the samples were assessed by AFM (NX10; AFM Park Systems, Suwon, Korea) and SEM (JSM-6500F system JEOL, Tokyo, Japan). Specimens were prepared by spin-coating diluted aqueous solutions onto silicon wafers at 1250 rpm for 15 s, and subsequently vacuum-dried at ambient temperature overnight.

### 2.4. Preparation of A-R6G/BU-PEG and R6G/BU-PEG Complexes

Different amounts of A-R6G or R6G (0.1 mg, 0.5 mg, and 1 mg) were added to BU-PEG (1 mg) in DMF (2 mL), stirred for 24 h, and then dialyzed against PBS (pH 7.4, 10 mM) or distilled water (DW) for 24 h (1000 Da molecular weight cut-off (MWCO)); the PBS (or DW) was replenished every 4 h. Finally, the absorption spectra of the A-R6G/BU-PEG solutions were obtained via UV/Vis spectrophotometry at λ = 525 nm to establish the concentration–absorbance standard curves of R6G and A-R6G. The absorption spectra of the A-R6G/BU-PEG and R6G/BU-PEG complexes (1 mg/mL) were compared to the standard curves for R6G and A-R6G. The following equation was used to calculate the drug loading content (DLC) and drug loading efficiency (DLE):

$$\mathrm{DLC}\%=\frac{\mathrm{Weight}\;\mathrm{of}\;\mathrm{drug}\;\mathrm{loaded}\;\mathrm{in}\;\mathrm{polymeric}\;\mathrm{nanogels}}{\mathrm{Weight}\;\mathrm{of}\;\mathrm{drug}\;\mathrm{loaded}\;\mathrm{polymeric}\;\mathrm{nanogels}\;}\;\times \;100$$

$$\mathrm{DLE}\%=\frac{\mathrm{Weight}\;\mathrm{of}\;\mathrm{drug}\;\mathrm{loaded}\;\mathrm{in}\;\mathrm{polymeric}\;\mathrm{nanogels}\;}{\mathrm{weight}\;\mathrm{of}\;\mathrm{drug}\;\mathrm{input}\;}\;\times \;100$$

### 2.5. Evaluation of the Stability of Pristine BU-PEG and R6G/A-R6G-Loaded BU-PEG Nanogels

The stability of blank and R6G/A-R6G-loaded BU-PEG nanogels in DMEM were investigated in the presence of FBS, which functions as a nanoparticle-destabilizing agent [34]. Pristine BU-PEG and A-R6G-loaded or R6G-loaded BU-PEG nanogels were mixed with DMEM containing 2:1 *v*/*v* serum, and the particle size and distributions were measured over 24 h by DLS.

### 2.6. Study of the Drug Release Behavior of Drug-Loaded Nanogels

The A-R6G-loaded or R6G-loaded BU-PEG nanogels (5 mL) were placed in dialysis tubing (MWCO = 1000 Da) and immersed in 50 mL of PBS with various pH values (7.4, 6.5, or 6.0) at 25 °C; the PBS was stirred (100 rpm) using a magnetic stirrer. At predefined intervals, 5 mL of external buffer solution was sampled and replaced with 5 mL of new PBS solution with the same pH. The amount of released R6G (or A-R6G) was measured by UV-Vis spectrometry at λ = 525 nm against a standard calibration curve for free R6G in PBS, and plotted vs. time.

### 2.7. Hemolysis Assay

Sheep red blood cells (SRBCs, Cosmo Bio, Tokyo, Japan) were used to assess the hemolytic activity of A-R6G-loaded and R6G-loaded BU-PEG nanogels. Briefly, 1 mL of the SRBCs and 0.5 mL of PBS were added into a microcentrifuge tube, centrifuged at 12,000 rpm for 15 min, and the supernatant was removed as plasma. Next, 1.5 mL of PBS was added, vortexed, and centrifuged; this wash step was repeated three times until the supernatant was clear. Subsequently, the resulting SRBC solutions (500 µL) were added to various concentrations of drug-loaded nanogels (10, 20, 40, 100, and 150 μg/mL). Triton X-100 solution (1%) was used as a positive control and PBS as a negative control. All samples were placed in a 5% CO_2_ incubator at 37 °C for 4 h, then centrifuged, and then 100 μL of the supernatants were transferred into a 96-well plate and the absorbance values were measured at a wavelength of 540 nm on an ELISA reader. The hemolysis index was calculated as follows:

$$\mathrm{Hemolysis}\%=\frac{A_{\mathrm{sample}}-A_{\mathrm{negative}}}{A_{\mathrm{positive}}-A_{\mathrm{negative}}}\;\times \;100\%$$

where a represents the optical density (OD) values of the test sample, positive control (1% Triton X-100), or negative control (PBS).

### 2.8. Cell Lines and Culture Conditions

HeLa cells (human cervical cancer cell lines), MG-63 (human bone cancer cell lines), and NIH/3T3 cells (mouse embryonic fibroblast cell lines) were obtained from the ATCC (American Type Culture Collection, Manassas, VA, USA) and routinely cultured in DMEM supplemented with 10% FBS containing 1% penicillin-streptomycin in T-75 culture flasks at 37 °C in a 5% CO_2_ incubator.

### 2.9. In Vitro Cell Cytotoxicity Assay

The cytotoxicity of the A-R6G-loaded and R6G-loaded BU-PEG nanogels were investigated against normal cell lines (NIH/3T3 cells) and cancer cell lines (HeLa and MG-63 cells) using the 3-(4,5-dimethylthiazol-2-yl)-2,5-diphenyltetrazolium bromide (MTT) method. Briefly, 1 × 10^5^ cells/well were seeded into 96-well plates in 100 μL of complete DMEM medium, allowed to adhere for 24 h, then the culture media were replaced with new media containing 0.1–100 μg/mL of blank BU-PEG, free R6G or A-R6G, or A-R6G-loaded or R6G-loaded BU-PEG nanogels. The plates were incubated for 24 h, the media were removed, and 100 μL of MTT assay solution (5 mg/mL) was added per well, incubated for 4 h, then 100 μL of dimethyl sulfoxide was added to dissolve the formazan crystals in each well. The optical densities were measured using an ELx800 microplate reader (BioTek, Winooski, VT, USA) at 570 nm.

### 2.10. Cellular Uptake of R6G/A-R6G-Loaded BU-PEG Nanogels

Intracellular uptake of A-R6G-loaded and R6G-loaded BU-PEG nanogels was evaluated in NIH/3T3 and HeLa cells by confocal laser scanning microscopy (CLSM) and flow cytometry.

CLSM: NIH/3T3 and HeLa cells were seeded into 35-mm microscopy dishes at a density of 1 × 10^5^, cultured for 24 h, then the medium was replaced with fresh serum-free medium containing A-R6G-loaded or R6G-loaded BU-PEG nanogels and incubated for various times (3, 12, or 24 h). Subsequently, the NIH/3T3 and HeLa cells were gently rinsed three times with cold PBS and fixed in 4% paraformaldehyde for 30 min at 25 °C. Thereafter, the nuclei of the cells were stained using 4′,6-diamidino-2-phenylindole (DAPI) for 30 min. The fluorescence of the cells was visualized via confocal microscopy (iRiS™ Digital Cell Imaging System, Logos Biosystems, Anyang-si, Korea).

Flow cytometry: NIH/3T3 and HeLa cells were seeded into 6-well plates at a density of 2 × 10^6^ cells/well, incubated for 24 h at 37 °C, then A-R6G-loaded or R6G-loaded BU-PEG nanogels dissolved in fresh free-serum medium were added to the wells and cultured for 1, 3, 6, 12, or 24 h. The cells were detached using trypsin, re-suspended with cold PBS (0.5 mL) and analyzed on a BD FACSAria III flow cytometer (BD Biosciences, San Jose, CA, USA). Data events were collected and determined by FlowJo software.

### 2.11. Analysis of Apoptosis Induced by R6G/A-R6G-Loaded BU-PEG Nanogels

The BV421-Annexin and Ghost Red Dye-780 Detection Kit was used to double stain the NIH/3T3 and HeLa cells. Briefly, the cells were seeded in 6-well plates at 1 × 10^6^ cells/well, allowed to adhere for 24 h, then incubated with A-R6G-loaded or R6G-loaded BU-PEG nanogels for different periods of time (1, 3, 6, or 12 h). Untreated NIH/3T3 and HeLa cells were prepared as controls. Next, the cells were re-suspended in binding buffer and stained using BV421-Annexin (15 min), followed by Ghost Red Dye-780 (15 min) in the dark, in accordance with the manufacturer’s protocol, then analyzed by flow cytometry (BD FACSAria III).

### 2.12. Statistical Analysis

All results are provided as the means and standard deviations of at least three independent experiments.

## 3. Results and Discussion

Herein, our research aims and objectives focused on the development of cooperative multiple hydrogen bonds in nucleobase-functionalized groups between a nanocarrier and drug molecules to enhance the safety and efficiency of cancer chemotherapy, as illustrated in Scheme 1. A new a water-soluble uracil-end-capped BU-PEG polymer was developed and was prepared via a one-step Michael addition reaction of PEGDA [32,33] to uracil using a *t*-BuOK catalyst, resulting in a high-yielding product (83%). The resulting BU-PEG polymer showed the desired chemical structures and molecular weight, as determined by ^1^H NMR, ^13^C NMR, and GPC (see Supplementary Materials, Figure S1). In addition, BU-PEG is a white, semi-crystalline powder and can easily dissolve in water at 25 °C, even at concentrations as high as 50 mg/mL. Due to the formation of A–U base-pairings between BU-PEG and adenine-functionalized molecules, a strong cooperative hydrogen bonding partner drug, A-R6G, was synthesized according to our previous report [31]. After the introduction of an adenine moiety into the R6G structure, the drug exhibited extremely poor solubility in water, buffer and biological media, demonstrated unique green-fluorescent behavior, and exerted strong cytotoxic effects in various normal and cancer cell lines [31,35,36,37]. Due to the significant differences in the water solubility between the BU-PEG polymer and A-R6G drug, these intriguing findings motivated our interest in exploring the co-assembly behavior of BU-PEG/A-R6G complexes in water.

**Figure 1:**
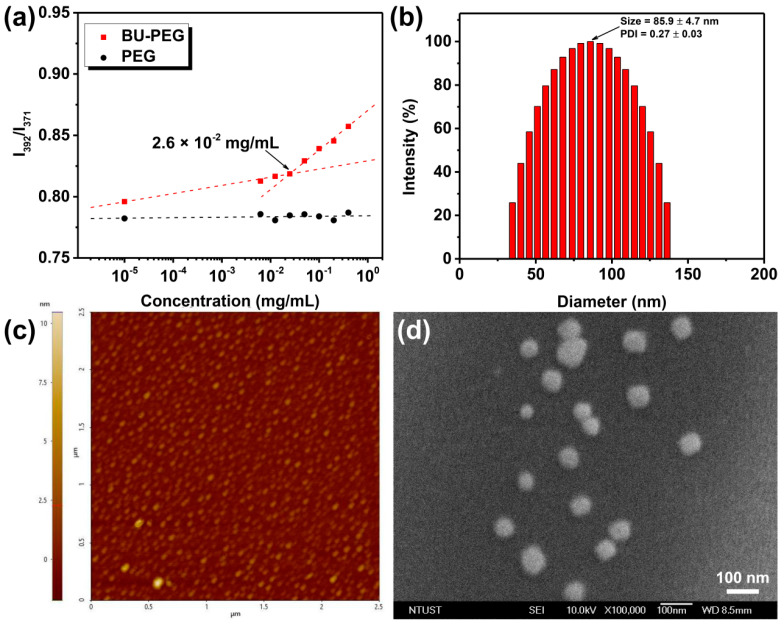
(**a**) Determination of the CMC values of PEG and BU-PEG in water in the presence of pyrene. (**b**) Hydrodynamic particle size of the BU-PEG polymer in water as determined by DLS at 25 °C. Surface morphologies of spin-coated BU-PEG polymers obtained by (**c**) AFM and (**d**) SEM at 25 °C.

Before discussing the hydrogen-bonded complexes of BU-PEG with A-R6G in water, we first determined the basic physical characteristics of BU-PEG in water to help our understanding and control the self-assembly structures and dynamics of BU-PEG in water and to guide the construction of a drug_carrier system based on the combination of BU-PEG and A-R6G. To investigate the effect of the hydrogen-bonded uracil groups on the amphiphilic features of the hydrophilic PEG backbone in aqueous environments, UV-Vis measurements using hydrophobic pyrene as a fluorescent probe were employed to determine the CMC values of pristine PEG (M_n_ = ~2000) and BU-PEG in water [38]. As shown in Figure 1a, BU-PEG showed a low CMC value of 2.6 × 10^−2^ mg/mL, whereas pristine PEG did not exhibit any CMC characteristics across a broad concentration range from 10^−5^ to 1 mg/mL, suggesting that the introduction of a uracil moiety into the PEG end-groups significantly impacted the amphiphilicity of the PEG backbone in water. This observation was possibly due to the presence of the self-complementary, interpolymer uracil hydrogen bonding interactions between the chains, which thus prompted the formation of relatively longer linear polymer chains connected by hydrogen-bonded uracil dimers that subsequently altered the overall amphiphilicity of BU-PEG. Next, we studied the self-organized structure of BU-PEG in water via complementary DLS, AFM and SEM measurements. When the concentration of the BU-PEG solution was 0.5 mg/mL (above its CMC value), BU-PEG had a mean hydrodynamic diameter of 86 nm, a PDI value of 0.27 and a *ζ*-potential value of −41.12 ± 0.36 mV (Figure 1b, Table S1), suggesting that BU-PEG could self-construct uniform, mono-distributed nanogels in water; this observation was attributed to the presence of the self-complementary uracil hydrogen bonding interactions within the polymer structure. In validation of the DLS results, AFM and SEM images confirmed that BU-PEG formed spherical nanogels with a diameter ranging from 35 to 70 nm (Figure 1c,d). Thus, these results revealed that the uracil units within the polymer structure served as a key governing force in hydrogen bonding molecular recognition to induce the self-assembly of the polymer in an aqueous environment.

After exploring the self-assembled structures and the characteristics of BU-PEG in water, we examined the molecular recognition of the A–U base-pairing between BU-PEG and A-R6G in D_2_O using ^1^H NMR spectroscopy. When a binary mixture of A-R6G and BU-PEG was prepared at a 1:10 blending weight ratio in D_2_O, the hydrophobic A-R6G almost completely dissolved in D_2_O and the mixed solution had a light orange color, while pristine A-R6G entirely precipitated in D_2_O, implying that A-R6G has a high tendency to interact with BU-PEG via strong complementary A–U interactions, thus leading to a significant increase in the solubility of A-R6G in D_2_O. Further ^1^H NMR analysis led to clear observation of the characteristic A-R6G peaks for the A-R6G/BU-PEG blend in D_2_O, whereas no peaks were detected for pristine A-R6G due to its poor water solubility (Figure S2). These results clearly and unequivocally demonstrate that the adenine units of A-R6G underwent complementary hydrogen bonding interactions with the uracil end-groups of BU-PEG in the aqueous solution, which thus promoted the formation of the A-R6G/BU-PEG complex and drastically improved the water-solubility of A-R6G. In addition, these results also implied that the existence of the complementary A–U interactions within the A-R6G/BU-PEG complexes may significantly improve the encapsulation capacity and stability of A-R6G after blending/mixing with BU-PEG. Thus, we subsequently performed an A-R6G encapsulation experiment to evaluate the drug-loading performance of BU-PEG nanogels via a dialysis method (see further details in the Experimental Section) in order to validate whether the complementary A–U interactions within the drug–carrier system successfully conferred high drug entrapment efficiency and improved drug-entrapment stability. As expected, the A-R6G-loaded BU-PEG complexes with a weight ratio of 1:1 had the maximal DLC of 69.04 ± 2.89%, whereas the R6G-loaded BU-PEG complexes with the same mixing ratio only achieved an R6G-loading content of 22.67 ± 3.45% (Table S1). Additionally, the resulting A-R6G-loaded BU-PEG complexes exhibited a wide-range tunable DLC, and the desired A-R6G-loading content could be achieved by controlling the A-R6G and BU-PEG blending ratio. In contrast to the A-R6G/BU-PEG system, the R6G and BU-PEG complexes prepared using different ratios of both materials exhibited similar DLC values after purification by dialysis (Table S1), indicating that R6G-loaded BU-PEG cannot stably encapsulate R6G due to the lack of complementary A–U interactions within the complexes, thus explaining the relatively low DLC and non-tailorable drug-loading content. In other words, the complementary hydrogen bonding interactions between uracil and the adenine moieties within the complexes increase the affinity and specificity of the nanocarriers for encapsulated hydrophobic drugs, and thus conferred extremely high and tunable DLC.

**Figure 2:**
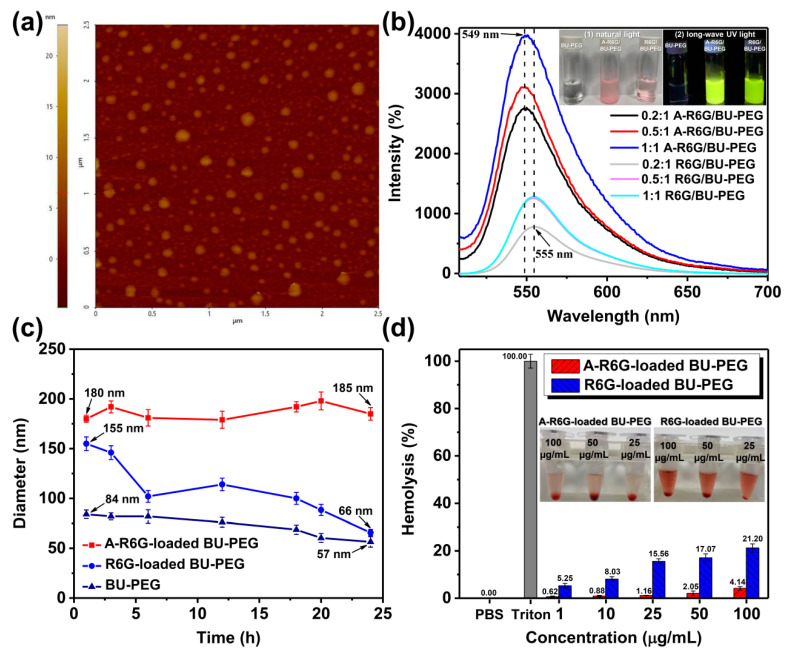
(**a**) AMF images of A-R6G-loaded BU-PEG nanogels measured at 25 °C. (**b**) PL spectra of A-R6G-loaded and R6G-loaded BU-PEG nanogels in water at 25 °C. Inset: photographs of A-R6G-loaded and R6G-loaded BU-PEG nanogels in water exposed to (1) natural lighting and (2) broadband UV lighting conditions. (**c**) Time-dependent DLS analysis of the structural stability of A-R6G-loaded and R6G-loaded BU-PEG nanogels in media containing 10% FBS at pH 7.4 for 24 h. (**d**) In vitro hemolytic assay of different concentrations of A-R6G-loaded and R6G-loaded BU-PEG nanogels on SRBCs. Inset: photographs showing SRBCs incubated with varying concentrations (1–100 μg/mL) of A-R6G-loaded or R6G-loaded BU-PEG nanogels at 37 °C in 5% CO_2_ for 4 h.

The DLS analysis further demonstrated that A-R6G-loaded BU-PEG (containing 69% A-R6G) and R6G-loaded BU-PEG (containing 23% R6G) displayed mean hydrodynamic diameters of 181 ± 5.97 nm (PDI = 0.461) and 160 ± 5.53 nm (PDI = 0.237), respectively (Figure S3a, Table S1), suggesting that the complexes increased in size to offer a relatively large capacity to accommodate a large number of drug molecules compared with the pristine BU-PEG nanogels. In addition, the *ζ*-potential value of the A-R6G-loaded BU-PEG increased progressively from −38.29 ± 6.44 mV to 51.21 ± 3.66 mV with a gradual increase of DLC. All of the A-R6G-loaded complexes exhibited significantly larger *ζ*-potential values compared to the R6G-loaded BU-PEG and the pristine BU-PEG (Table S1), indicating the *ζ*-potential value of the A-R6G-loaded BU-PEG complexes gradually increased to improve the A-R6G encapsulation efficiency and stability of the complexes. In order to verify the results obtained by DLS, we examined the morphological structure of A-R6G-loaded and R6G-loaded BU-PEG complexes using AFM and SEM. As illustrated in Figure 2a and Figure S3b–d, both systems had nearly spherical shapes with sizes varying between 120–150 nm and 90–130 nm, respectively, consistent with the DLS results (Figure S3a). Overall, these findings further revealed that dynamic complexes of BU-PEG with hydrophobic A-R6G suspended in water were successfully constructed due to the complementary A–U interactions, and resulted in formation of a spherical-like A-R6G-encapsulated nanogel with tunable DLC capacity.

Due to the intrinsic fluorescence emission of R6G and A-R6G, we further investigated the effect of A–U base pairing on the fluorescence properties of the A-R6G-loaded and R6G-loaded BU-PEG nanogels in water with different DLC values using PL spectroscopy. As shown in the right-upper inset of Figure 2b, when A-R6G and the BU-PEG were blended at a 1:1 weight ratio, the resulting aqueous solution (containing 51% A-R6G) exhibited a strong, bright-green fluorescence under excitation with a broadband UV, while the R6G-loaded BU-PEG solution (weight ratio of 1:1; containing 19% R6G) exhibited relatively weak green fluorescence. These results indicated the complementary A–U interactions within the nanogels dramatically enhanced the fluorescence emission behavior of A-R6G in aqueous solution. A quantitative analysis of the fluorescence enhancement of A-R6G-loaded BU-PEG nanogels in water was conducted by PL measurements with excitation at 480 nm. As shown in Figure 2b, the PL spectra of R6G-loaded BU-PEG nanogels containing 13% and 19% R6G (weight ratios of 1:1 and 0.5:1, respectively) in water exhibited maximum fluorescence peaks at 555 nm with the same intensity of around 1300, possibly due to the presence of the similar DLC of the nanogels. Interestingly, the maximum PL fluorescence peaks and intensities of the A-R6G-loaded BU-PEG nanogels with various DLC values were remarkably different to those of the R6G-loaded BU-PEG system: the A-R6G-loaded nanogels exhibited a significant blue-shift in their maximum fluorescence peaks from 555 nm to 549 nm, and a gradual increase in the maximum fluorescence intensity from 2740 to 3980 as the DLC increased, suggesting that the physical encapsulation of the A-R6G in the nanogels through complementary A–U interactions efficiently prevented the aggregation of the polycyclic aromatic rings of A-R6G within the nanogels [39,40], resulting in a significant blue-shift of the maximum fluorescence peak and a progressive enhancement in fluorescence intensity with the DLC, even with A-R6G-loading contents as high as 51%. Thus, these findings further demonstrate that the A–U interactions within this complementary drug-nanocarrier system manipulate the drug-encapsulation and fluorescence behavior of the nanogels in aqueous environments.

An ideal nanoparticulate drug-loaded delivery system must maintain high drug-entrapment stability in the normal cellular environment to ensure safe, effective delivery of drugs. Therefore, we studied the structural stability of A-R6G-loaded and R6G-loaded BU-PEG nanogels in culture media (DMEM supplemented with 10% serum FBS) and the SRBC hemolysis assay. FBS and SRBCs were employed as nanoparticle-destructuring agents to induce the rapid disassembly of the self-assembled nanogels [34]. As shown in Figure 2c, pristine BU-PEG exhibited a gradual decrease in the mean hydrodynamic diameter from 84 nm to 57 nm after 24 h incubation with FBS-containing DMEM, implying that the self-complementary hydrogen bonding uracil moieties could not preserve the structural integrity of the BU-PEG nanogels in FBS/PBA-mixed medium. After the encapsulation process, A-R6G-loaded BU-PEG nanogels remained at an almost constant mean hydrodynamic diameter after 24 h monitoring, whereas a progressive decrease in the particle size of the R6G-loaded BU-PEG nanogels from 155 nm to 66 nm was clearly observed, indicating the stable complementary A–U interactions between the A-R6G and the BU-PEG complex substantially improved drug-retention stability and prevented initial drug leakage from the nanogels. Similar trends in the improvement of structural stability were obtained in the hemolysis assay. As presented in Figure 2d and the inset photographs, the SRBC hemolytic assay indicated that a broad range of concentrations (1–100 μg/mL) of A-R6G-loaded BU-PEG nanogels did not exert significant hemolytic activity. Even at high concentrations up to 100 μg/mL, the A-R6G-loaded nanogels showed a low hemolytic activity of 4.1%, indicating the excellent compatibility of the nanogels with blood, which is potentially favorable for in vivo applications [41,42]. In contrast to the A-R6G-loaded BU-PEG system, the percentage hemolysis gradually increased as the concentration of R6G-loaded BU-PEG nanogels increased. The percentage hemolysis was up to 21.2% at 100 μg/mL of R6G-loaded BU-PEG, indicating that BU-PEG nanogels cannot maintain their structural stability due to the lack of complementary interactions between BU-PEG and R6G, thus resulting in significant hemolysis. The combination of long-term drug-entrapment stability and low-level hemolysis for A-R6G-loaded BU-PEG is an extremely attractive set of features that are rarely observed in traditional drug carrier systems. Therefore, A-R6G-loaded BU-PEG nanogels may represent a potential drug-delivery system that can provide a safe, reliable and efficient delivery of A-R6G within cellular environments.

**Figure 3:**
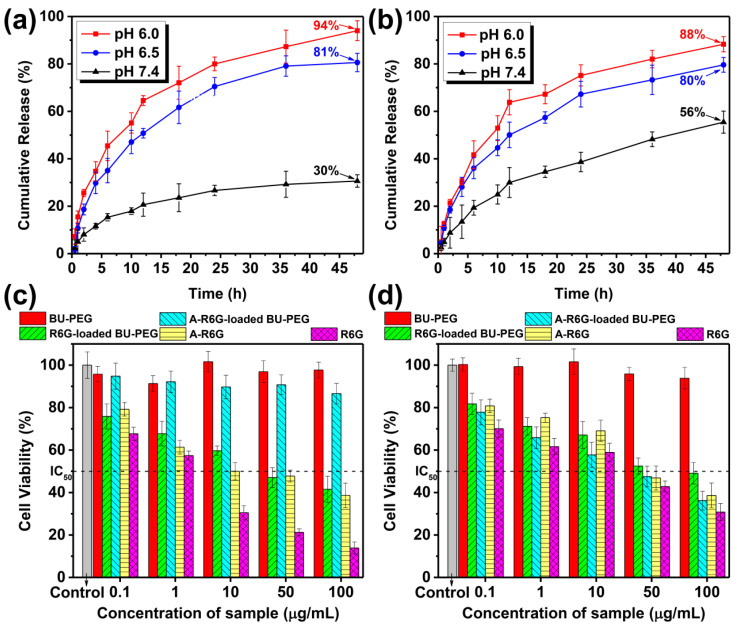
In vitro drug release profiles of (**a**) A-R6G-loaded and (**b**) R6G-loaded BU-PEG nanogels. In vitro cytotoxicity of A-R6G-loaded and R6G-loaded BU-PEG nanogels against (**c**) NIH/3T3 cells and (**d**) HeLa cells after 24 h incubation.

The above findings prompted us to further evaluate the drug release behavior of A-R6G (or R6G) from BU-PEG nanogels in PBS solutions at different pH values (pH 7.4, 6.5, 6.0) at 25 °C using a dialysis method. At pH 7.4, the cumulative release of A-R6G from BU-PEG nanogels was only 30% after 48 h, whereas R6G release of up to 56% was observed from the BU-PEG nanogels, suggesting that the encapsulated A-R6G within the nanogels exhibited high structural stability under normal physiological conditions due to the complementary A–U interactions, thus leading to slow drug release and low cumulative drug release (Figure 3a,b). Interestingly, when the environmental pH was decreased to 6.5 or 6.0, the A-R6G or R6G was released much more quickly than at a normal physiological pH of 7.4. Both systems exhibited a significant initial burst, with over 50% of the drug released in the first 12 h at either pH 6.5 or 6.0, followed by cumulative release of 94% and 88% after 48 h, respectively, implying that weakly acidic environments can trigger the structural disassembly of both drug-loaded nanogels, and thus induce a rapid drug release rate and high cumulative drug release. Overall, due to the transient A–U interactions within their structure, A-R6G-loaded BU-PEG nanogels exhibit high A-R6G-loading capacity and excellent structural stability that directly inhibits premature drug release and a confers a low drug release rate under normal physiological pH 7.4. However, the A-R6G-loaded BU-PEG nanogels disassembled and release the drug rapidly in mildly acidic conditions, and thereby possessed well-controlled pH-triggered drug release properties. Thus, this self-assembled drug–carrier system based on complementary A–U interactions may represent an attractive and potential route for safer, more efficient in vitro and in vivo delivery and release of medication.

Potential drug delivery nanogels must be highly biocompatible and exert low cytotoxicity against normal and cancer cells, and they must also only release the encapsulated drug under specific conditions in the cellular environment. Thus, an MTT-based chromogenic assay was used to quantify the cytotoxic activity of pristine BU-PEG, A-R6G-loaded-, and R6G-loaded BU-PEG nanogels toward normal NIH/3T3 cells and cancerous HeLa and MG-63 cells. As indicated in Figure 3c,d and Figure S4, a range of concentrations of BU-PEG nanogels exerted negligible cytotoxic effects in normal and cancer cells after 24 h, indicating the BU-PEG was highly biocompatible. In contrast, pristine the A-R6G and R6G showed highly potent cytotoxic activities against normal and cancer cells, with half-maximal inhibitory concentrations (IC_50_) ranging from 1 to 43 µg/mL. Extraordinarily, after culture with A-R6G-loaded BU-PEG nanogels at concentrations up to 100 µg/mL for 24 h, the viability of NIH/3T3 cells remained above 85%, while the R6G-loaded BU-PEG nanogels strikingly reduced the viability of NIH/3T3 cells, with an IC_50_ value of 40.5 μg/mL (Figure 3c). These observations clearly suggested the complementary A–U interactions within the A-R6G-loaded BU-PEG nanogels significantly improved the structural stability of the nanogels and minimized premature A-R6G leakage under physiological conditions in NIH/3T3 cells. Conversely, the lack of complementary interactions within the R6G-loaded nanogels led to a significant reduction in NIH/3T3 cell viability. However, both the A-R6G-loaded and R6G-loaded BU-PEG nanogels exerted significant cytotoxic activities against HeLa and MG-63 cancer cells, with remarkable IC_50_ values of 39.7 μg/mL and 79.3 μg/mL in HeLa cells and 2.8 μg/mL and 9.3 μg/mL in MG-63 cells, respectively (Figure 3d and Figure S4). These results suggest that the pH-induced structural disassembly of both drug-loaded nanogels in the acidic extracellular environment of cancer cells led to rapid drug release, subsequently resulting in selective cytotoxic effects [43,44]. Thus, the weakly acidic extracellular cancer cell environment may facilitate the release of A-R6G within the interior of the cells and thus facilitate selective, potent cytotoxicity toward cancer cells, while reducing the adverse impacts of A-R6G-loaded nanogels in normal cells. Therefore, A-R6G-loaded BU-PEG nanogels could potentially significantly enhance the chemotherapeutic safety and effectiveness of anticancer drugs. However, the exact mechanisms of action of the selective cytotoxicity of A-R6G-loaded BU-PEG nanogels still remain uncertain. We are conducting research to more precisely define the structural features and in vivo cytotoxic effects of this drug–carrier system in order to confirm the selective and targeted cytotoxic effects of A-R6G towards cancer cells for chemotherapy applications.

**Figure 4:**
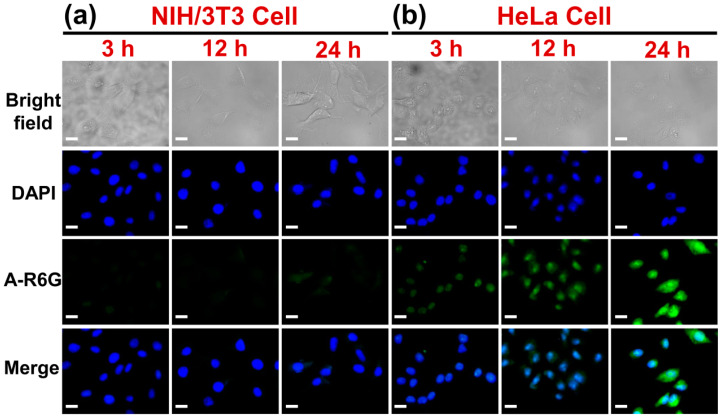
CLSM images of (**a**) NIH/3T3 cells and (**b**) HeLa cells cultured with A-R6G-loaded BU-PEG nanogels at normal physiological conditions (pH 7.4 and 37 °C) for 3, 12, or 24 h. The scale bars in all CLSM images are 20 µm.

In order to obtain further insight into the mechanisms of cellular uptake and intracellular drug release by A-R6G-loaded BU-PEG towards normal and cancer cells, NIH/3T3 and HeLa cells cultured with the drug-loaded nanogels for 3, 12, or 24 h, and then analyzed by CLSM to observe the cellular morphology and internalization of the nanogels [45]. Blue-fluorescent DAPI was used to stain the nuclei; A-R6G and R6G exhibit strong green fluorescence emission. As indicated in Figure 4a,b, the CLSM images clearly indicated no significant green fluorescent could be observed after 24 h incubation of NIH/3T3 cells with A-R6G-loaded BU-PEG nanogels. In contrast, remarkable green fluorescence was randomly distributed throughout the cytoplasm of the HeLa cells after 3 h incubation with A-R6G-loaded nanogels, and the green fluorescent signal gradually shifted into the nucleus after 24 h of incubation. In contrast to the A-R6G-loaded BU-PEG nanogels, a gradual increase in the green fluorescent signal and intensity was observed within the nuclei of NIH/3T3 or HeLa cells incubated with R6G-loaded BU-PEG nanogels for 3 to 24 h (Figure S5a,b). These findings are in good agreement with the MTT assay, and suggest the A-R6G-loaded BU-PEG nanogels were selectively internalized by the cancer cells and did not internalize in the normal cellular environment [46]. In contrast, the R6G-loaded BU-PEG nanogels underwent intensive, non-specific uptake by normal and cancer cells, possibly due to specific interactions between the A–U base-paring moieties of the nanogels and the surface of cancer cells. Overall, the above-mentioned findings indicate this supramolecular drug–carrier system containing complementary A–U pairs could promote the selective uptake of drugs by cancer cells and effectively induce cancer cell death while minimizing the cytotoxicity in normal cells.

**Figure 5:**
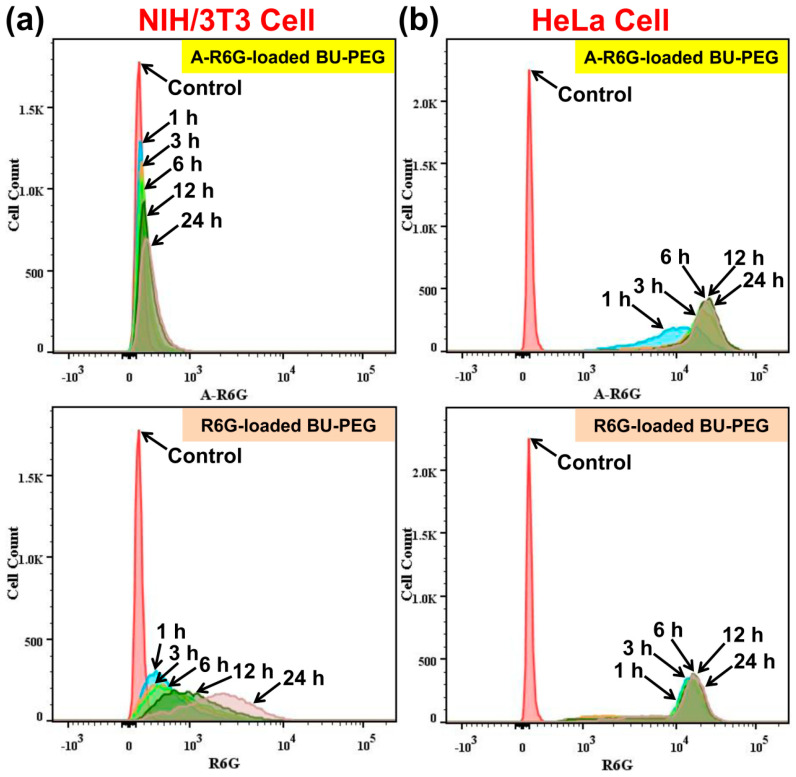
Flow cytometry histograms of (**a**) NIH/3T3 cells and (**b**) HeLa cells cultured with A-R6G-loaded or R6G-loaded BU-PEG nanogels at normal physiological conditions (pH 7.4 and 37 °C) for 1, 3, 6, 12, or 24 h.

In order to verify the CLSM images, we performed quantitative and qualitative flow cytometry analysis to further investigate the selective internalization of A-R6G-loaded BU-PEG nanogels by HeLa cells. As shown in Figure 5a,b, after incubation with A-R6G-loaded BU-PEG nanogels for 24 h, NIH/3T3 cells exhibited almost no change in A-R6G fluorescence intensity, whereas the A-R6G fluorescence intensity of HeLa cells gradually increased with the duration of incubation, indicating the complementary A–U base-pairs within the nanogels were endowed with a strong affinity for HeLa cells and thereby promoted selective, rapid internalization of the nanogels and subsequently led to the death of the cancer cells. In contrast, flow cytometry of NIH/3T3 and HeLa cells incubated with R6G-loaded BU-PEG nanogels for various periods of time revealed a gradual increase in R6G fluorescence intensity between 1 and 24 h, suggesting that the R6G/BU-PEG system was not able to selectively promote the internalization of nanogels by cancer cells. In addition, these results also revealed that R6G-loaded BU-PEG nanogels exhibited much higher and faster cellular uptake in HeLa cells than in NIH/3T3 cells (the lower left and right regions of Figure 5), possibly due to the differences in the surface charge and affinity between the cells. Moreover, we further assessed how the complementary hydrogen bonding interactions between the drug and carrier affected cellular uptake ability by plotting the average fluorescence intensity of the flow cytometry data versus incubation time. As shown in Figure S6, the internalization rate of the A-R6G-loaded BU-PEG nanogels was approximately 74 times higher in HeLa cells than NIH/3T3 cells after 24 h, whereas the internalization rate of the R6G-loaded BU-PEG nanogels was only 5.7 times higher in HeLa cells than NIH/3T3 cells after 24 h, which is in good agreement with the CLSM results (Figure 4 and Figure S5). These results further demonstrate that the A-R6G-loaded BU-PEG nanogels were selectively internalized into the HeLa cells but only minimally taken up by NIH/3T3 cells, whereas the R6G-loaded BU-PEG nanogels were non-specifically internalized by cells. Collectively, these findings clearly prove the complementary A–U interactions within the nanogels not only controlled their drug delivery and release properties, but also promoted the selective uptake of drugs into cancer cells and accelerated cell death; they may thus potentially enhance the overall efficiency of chemotherapy.

**Figure 6:**
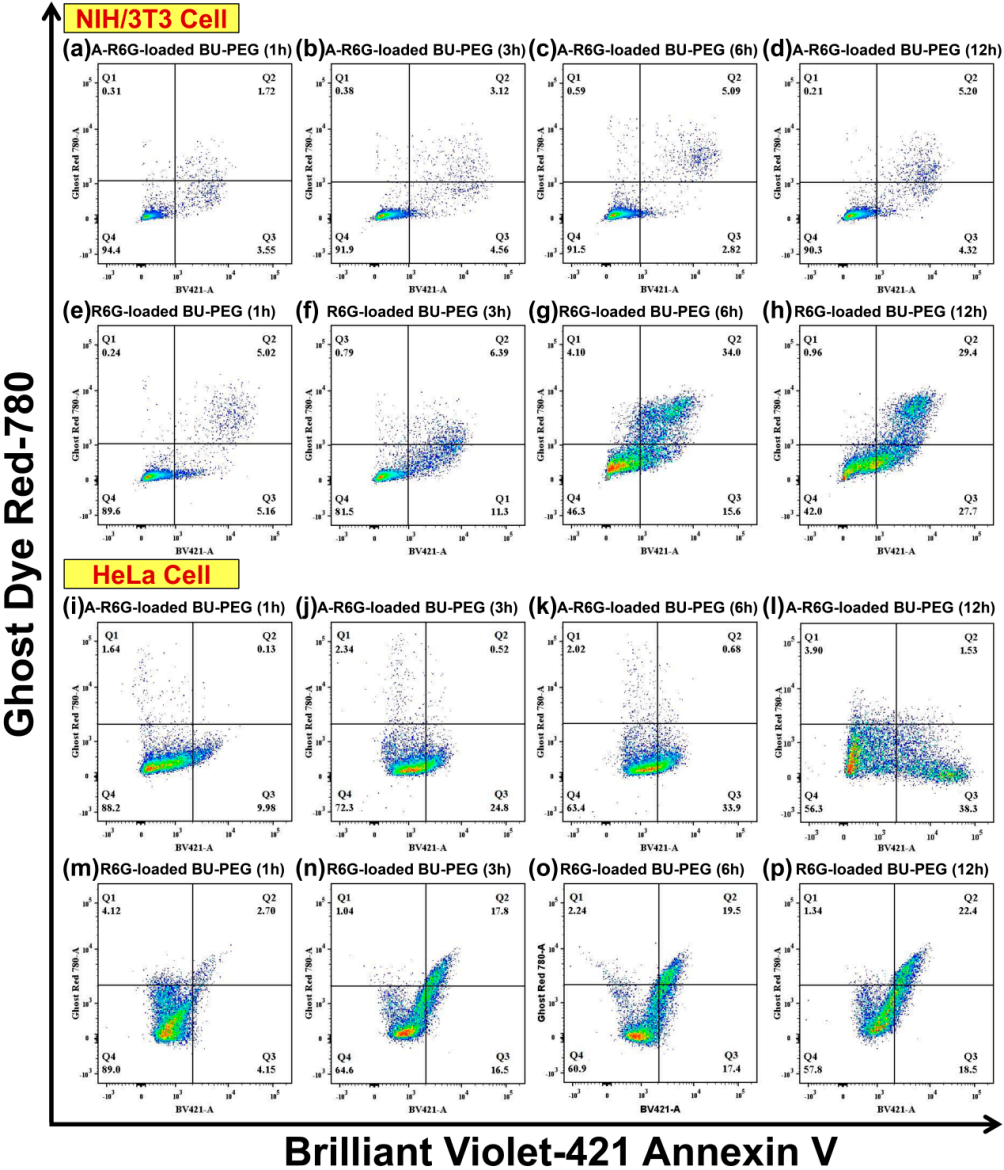
Flow cytometric dot plot quadrant charts of NIH/3T3 and HeLa cells cultured with A-R6G-loaded and R6G-loaded BU-PEG nanogels at normal physiological conditions (pH 7.4 and 37 °C) for 1, 3, 6, or 12 h before staining with BV421 Annexin V and GDR780. Figures (**a**)–(**p**) represent the results of flow cytometry for different time points during the co-culture period. The graph quadrants from the lower left to the upper left (turning anti-clockwise) represent viable cells (BV421 Annexin V^-^, GDR780^−^), early apoptotic cells (BV421 Annexin V^+^, GDR780^−^), late apoptotic cells (BV421 Annexin V^+^, GDR780^+^), and necrotic cells (BV421 Annexin V^−^, GDR780^+^). The numbers inside each quadrant refer to the proportions of cells.

To further identify the cytotoxic pathways and assess the mechanisms of cell death for A-R6G-loaded and R6G-loaded BU-PEG nanogels, dual fluorescent staining and flow cytometry were used to quantify viable, dead, and total cells after exposure of NIH/3T3 and HeLa cells to A-R6G-loaded and R6G-loaded BU-PEG nanogels for various periods of time. BV421 Annexin-V was used to detect phosphatidylserine expression on early apoptotic cells, while GDR-780 was used to label intracellular DNA, which is released after the integrity of the plasma membrane has been compromised in late apoptotic cells [47,48]. As shown in Figure 6d,h, after the incubation of HeLa cells with A-R6G-loaded or R6G-loaded BU-PEG nanogels for 12 h, respectively, over 90% of the NIH/3T3 cells incubated with A-R6G-loaded nanogels survived, whereas the R6G-loaded nanogels increased the proportions of early and late apoptotic cells to 27.7% and 29.4%, respectively, indicating that the A-R6G-loaded BU-PEG nanogels had extremely stable drug entrapment stability in a normal cellular environment, which significantly reduced the leakage of hydrophobic A-R6G during the drug delivery process. In contrast to the general trend in NIH/3T3 cells, the overall proportion of apoptotic HeLa cells gradually increased between 1 and 12 h, suggesting that the A-R6G-loaded nanogels taken up by the HeLa cells moved progressively toward the nucleus, and that the A-R6G was gradually released from the nanogels (Figure 6i–l). After 12 h of incubation with A-R6G-loaded nanogels, the proportions of early and late apoptotic HeLa cells dramatically increased to 38.3% and 1.53%, respectively, while the proportion of viable cells still remained high, at 56% (Figure 6l). By comparison, the apoptotic trends in the HeLa cells cultured with R6G-loaded BU-PEG nanogels were similar to the results for the NIH/3T3 cells presented in Figure 6h,p. These observations clearly confirm that the mildly acidic cancer microenvironment promotes the rapid intracellular release of A-R6G from BU-PEG by inducing dissociation of the complementary A–U interactions within the nanogels, and that the A-R6G subsequently promotes programmed cell death. Thus, this newly developed system based on complementary hydrogen bonding A–U interactions between A-R6G and BU-PEG may potentially remarkably enhance the effects of chemotherapy in cancer cells while substantially reducing adverse effects in healthy cells.

## 4. Conclusions

We successfully developed a complementary drug delivery system based on cooperative hydrogen bonding A–U interactions between a drug and nanocarrier to achieve selective uptake by cancer cells, improve chemotherapeutic efficacy, and reduce adverse effects in normal cells. A R6G-based anticancer agent containing a hydrogen-bonded adenine unit (A-R6G) was obtained via a simple three-step chemical reaction. A-R6G displays poor aqueous solubility and unique green fluorescent behavior, and exerts potent cytotoxic effects against a variety of cell lines. A complementary hydrogen bonding partner, water-soluble uracil-end-capped BU-PEG polymer, was prepared at high yield (83%) via a one-step Michael addition reaction. BU-PEG can spontaneously self-assemble into well-defined nanoparticles with a variety of unique physical properties in water, such as interesting amphiphilic and morphological characteristics. The formation of A–U base pairs between BU-PEG and A-R6G leads to the formation of well-dissolved A-R6G-loaded BU-PEG nanogels in water. Interestingly, the resulting A-R6G-loaded BU-PEG nanogels exhibited a number of unique physical properties, including extremely high A-R6G-loading capacity (69.4%), a widely tunable A-R6G-loading content, singular green-fluorescence characteristics, and well-controlled pH-responsive drug-release behavior. Moreover, A-R6G-loaded BU-PEG nanogels showed excellent structural stability in cell culture media and low hemolytic activity towards SRBCs. The combination of long-term drug-entrapment stability and low-level hemolytic activity offered by A-R6G-loaded BU-PEG is extremely attractive, but rare in traditional drug–carrier systems. In vitro cytotoxicity studies clearly confirmed that A-R6G-loaded BU-PEG nanogels exhibit potent cytotoxic activity against cancerous HeLa and MG-63 cells, and only minimal cytotoxic effects in normal NIH/3T3 cells, whereas R6G-loaded BU-PEG nanogels did not show selective cytotoxicity against these cell lines. Thus, the complementary A–U interactions critically improve the selective uptake of A-R6G into cancer cells and selectively induce cancer cell death. Importantly, analysis of intracellular cellular uptake using CLSM and flow cytometry clearly demonstrated that A-R6G-loaded BU-PEG nanogels enabled the selective uptake of A-R6G by HeLa cells via endocytosis and promoted controlled intracellular release of A-R6G in the weakly acidic microenvironment of the cancer cells, which subsequently induced programmed cell death in the cancer cells. The opposite trends were observed in NIH/3T3 cells, i.e., poor internalization and extremely low toxicity. Thus, this study clearly demonstrates that introduction of complementary A–U interactions within this drug–carrier system provides an effective approach to selectively delivering anticancer drugs into cancer cells, subsequently improving the safety and effectiveness of chemotherapy, without the need to incorporate targeting moieties onto the surface of drug carriers.

## Data Availability

Not applicable.

## Supplementary Materials

The following are available online at https://www.mdpi.com/article/10.3390/pharmaceutics13111929/s1, Scheme S1: Process for the synthesis of BU-PEG, Figure S1: (**a**) ^1^H and ^13^C NMR spectra of BU-PEG in CDCl_3_ obtained at 25 °C. (**b**) GPC curves for PEG and BU-PEG in THF at 40 °C, Figure S2: ^1^H NMR spectra of BU-PEG, A-R6G, and A-R6G/BU-PEG complexes in D_2_O obtained at 25 °C, Figure S3: (**a**) DLS profiles for A-R6G-loaded and R6G-loaded BU-PEG nanogels in water at 25 °C. (**b**) AFM image of R6G-loaded BU-PEG. SEM images of (**c**) A-R6G-loaded BU-PEG and (**d**) R6G-loaded BU-PEG nanogels, Figure S4: In vitro MTT assay cytotoxicity of BU-PEG, A-R6G, R6G, and A-R6G-loaded and R6G-loaded BU-PEG nanogels towards MG-63 cells, Figure S5: CLSM images of (**a**) NIH/3T3 cells and (**b**) HeLa cells cultured with R6G-loaded BU-PEG nanogels at normal physiological conditions (pH 7.4 and 37 °C) for 3 h, 12 h, or 24 h. The scale bars in all CLSM images are 20 µm, Figure S6: Fluorescence intensity of NIH/3T3 and HeLa cells after incubation with A-R6G-loaded and R6G-loaded BU-PEG nanogels for different periods of time (1, 3, 6, 12, or 24 h), Table S1: Hydrodynamic particle size, zeta potential, drug-loading content (DLC) and drug-loading efficiency (DLE) of A-R6G-loaded and R6G-loaded BU-PEG nanogels.
