# Supplementary material for: Complementary Nucleobase Interactions Drive Co-Assembly of Drugs and Nanocarriers for Selective Cancer Chemotherapy
Source: Pharmaceutics. 2021 Nov 15;13(11):1929. doi: 10.3390/pharmaceutics13111929 (PMC8625492; doi:10.3390/pharmaceutics13111929)
Supplement: Supplementary file 1 [file pharmaceutics-13-01929-s001.zip › pharmaceutics-1448187-supplementary.pdf]

pharmaceutics

Supplementary Materials: Complementary Nucleobase Interactions Drive Co-Assembly of Drugs and Nanocarriers for Selective Cancer Chemotherapy

Fasih Bintang Ilhami, Enyew Alemayehu Bayle and Chih-Chia Cheng

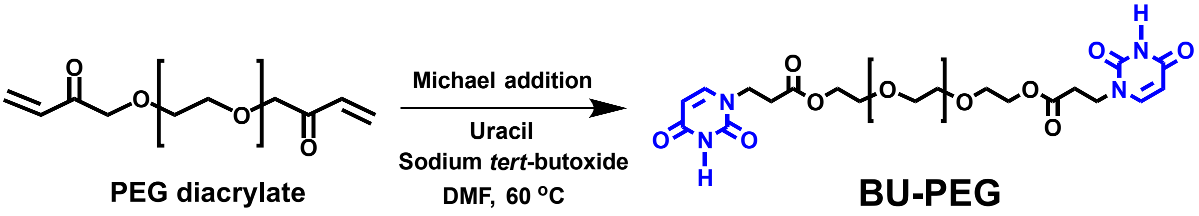

Scheme S1. Process for the synthesis of BU-PEG.

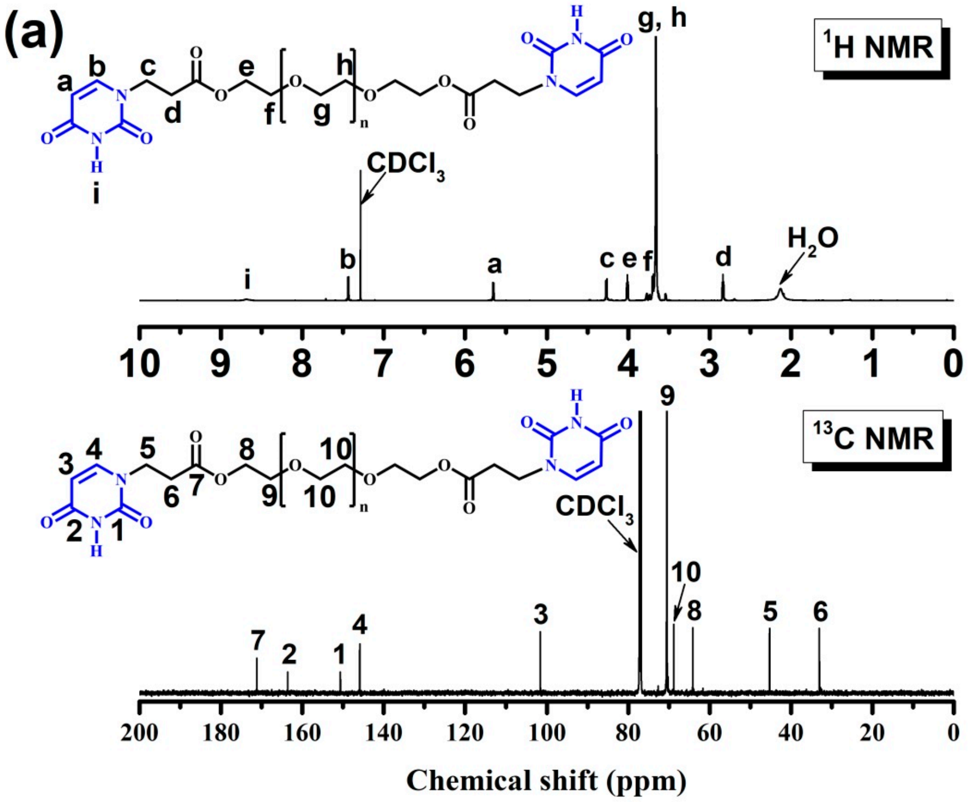

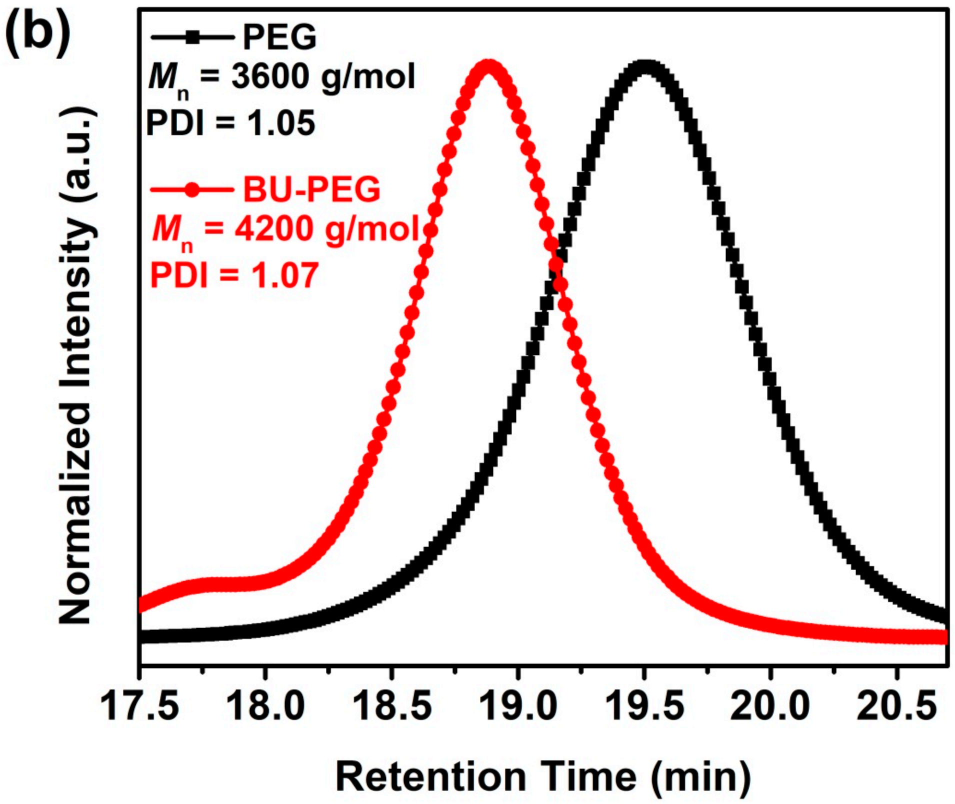

Figure S1. (a) ^{1}H and ^{13}C NMR spectra of BU-PEG in $CDCl_3$ obtained at 25 °C. **(b)** GPC curves for PEG and BU-PEG in THF at 40 °C.

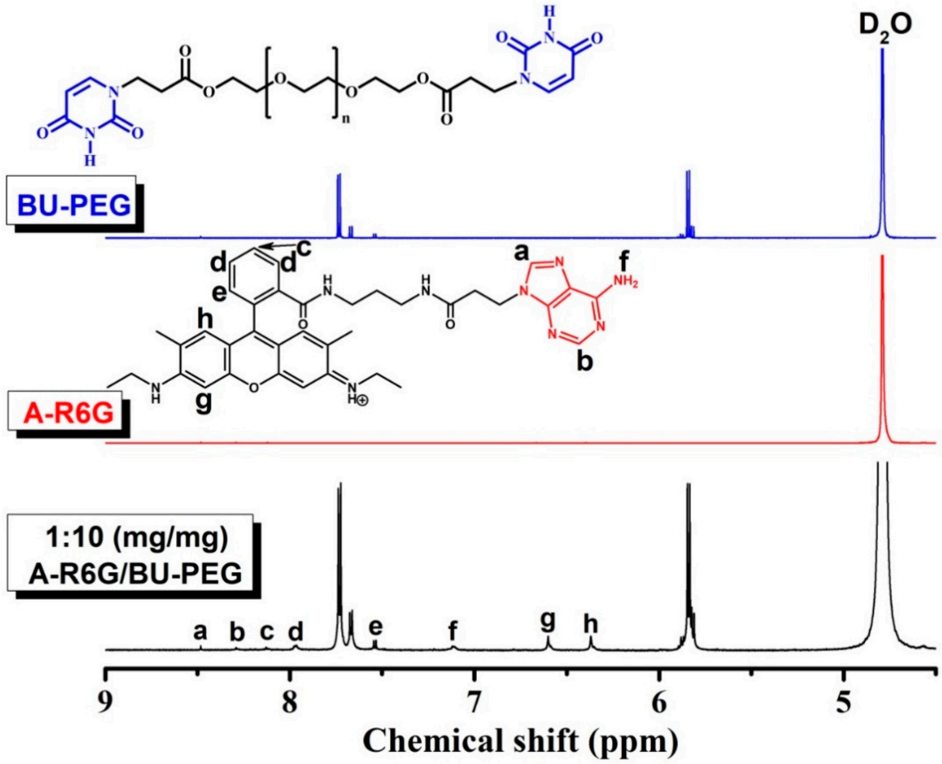

Figure S2. ^{1}H NMR spectra of BU-PEG, A-R6G, and A-R6G/BU-PEG complexes in D_2O obtained at 25 °C.

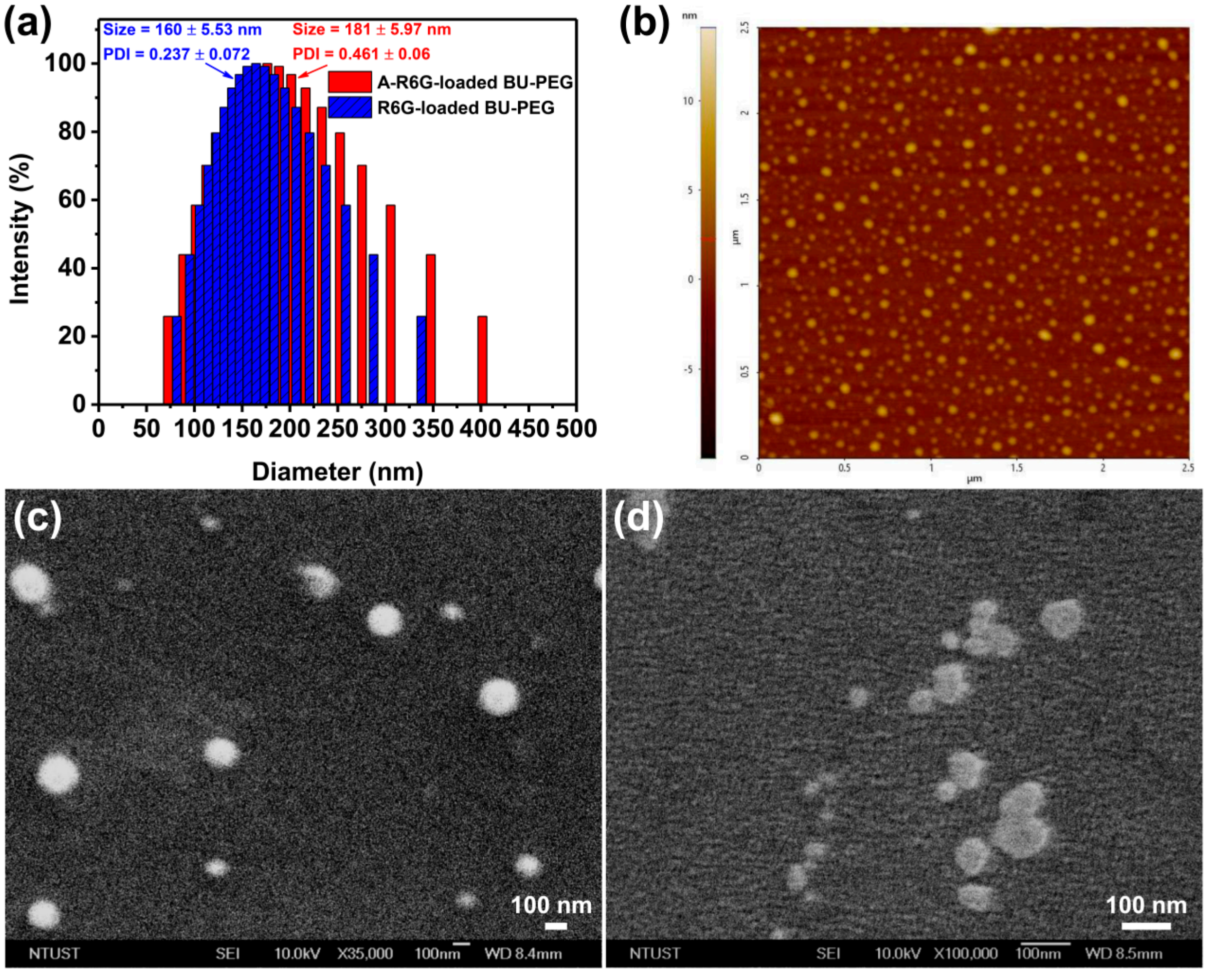

Figure S3. **(a)** DLS profiles for A-R6G-loaded and R6G-loaded BU-PEG nanogels in water at 25 °C. **(b)** AFM image of R6G-loaded BU-PEG. SEM images of **(c)** A-R6G-loaded BU-PEG and **(d)** R6G-loaded BU-PEG nanogels.

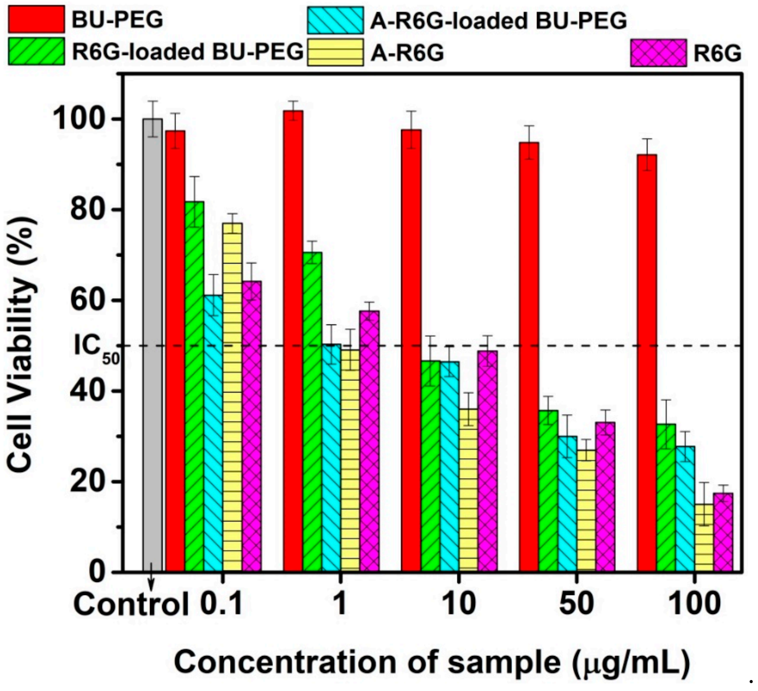

Figure S4. *In vitro* MTT assay cytotoxicity of BU-PEG, A-R6G, R6G, and A-R6G-loaded and R6G-loaded BU-PEG nanogels towards MG-63 cells.

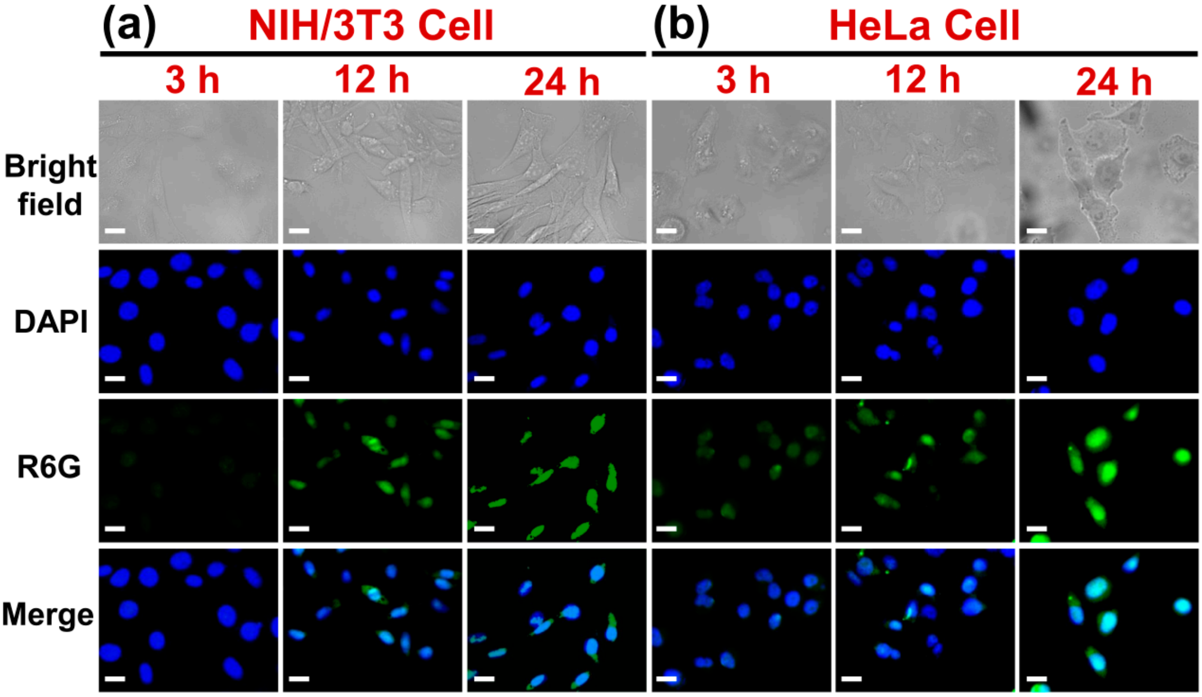

Figure S5. CLSM images of **(a)** NIH/3T3 cells and **(b)** HeLa cells cultured with R6G-loaded BU-PEG nanogels at normal physiological conditions (pH 7.4 and 37 °C) for 3 h, 12 h, or 24 h. The scale bars in all CLSM images are 20 μm.

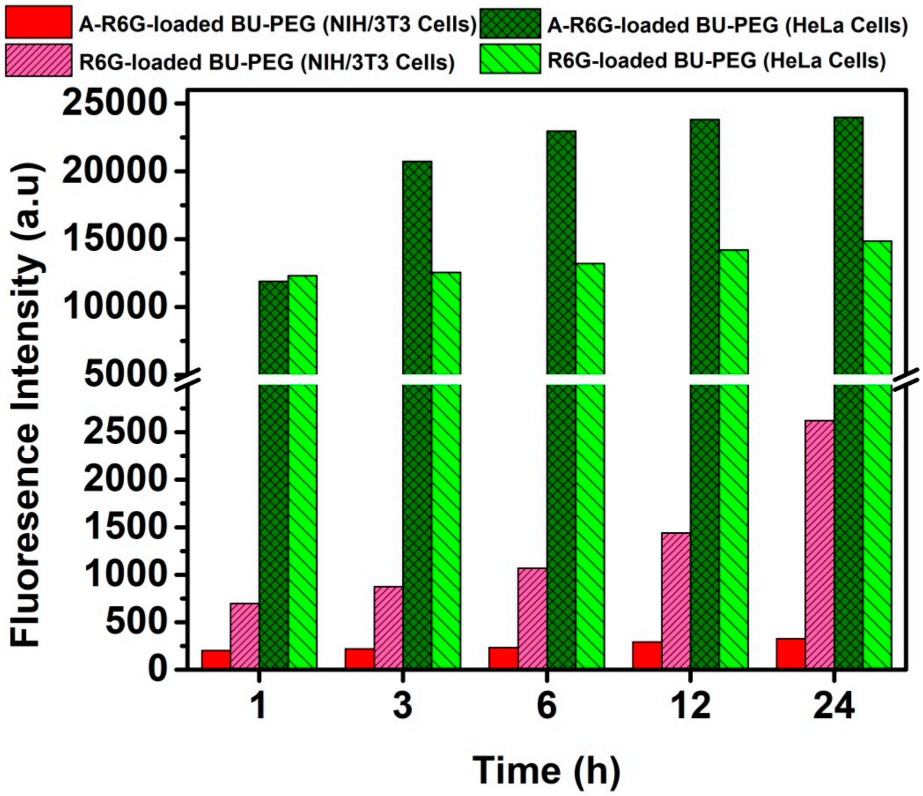

Figure S6. Fluorescence intensity of NIH/3T3 and HeLa cells after incubation with A-R6G-loaded and R6G-loaded BU-PEG nanogels for different periods of time (1, 3, 6, 12, or 24 h).

Table S1. Hydrodynamic particle size, zeta potential, drug-loading content (DLC) and drug-loading efficiency (DLE) of A-R6G-loaded and R6G-loaded BU-PEG nanogels.

A-R6G-loaded sample	Drug:Carrier (weight ratio)	Particle Size	Zeta Potential (mV)	DLC ± SD (%)	DLE ± SD (%)
BU-PEG	-	85.9 ± 4.67	4.31 ± 4.47	-	-
A-R6G/BU-PEG	0.2:1	102 ± 6.13	−38.29 ± 6.44	37.19 ± 1.76	55.23 ± 4.15
	0.5:1	148 ± 5.09	−40.37 ± 5.95	55.50 ± 1.81	46.83 ± 1.30
	1:1	181 ± 5.97	−51.21 ± 3.66	69.04 ± 2.89	33.76 ± 1.48
R6G/BU-PEG	0.2:1	108 ± 4.93	12.47 ± 6.94	18.45 ± 1.77	70.32 ± 2.45
	0.5:1	123 ± 7.58	13.32 ± 6.04	20.14 ± 2.07	59.83 ± 1.46
	1:1	160 ± 5.53	19.29 ± 5.11	22.67 ± 3.45	43.71 ± 4.43
